# Factors Influencing Orchid Species Richness in the Central Balkans: The Importance of Belowground Organ Types

**DOI:** 10.3390/plants14030443

**Published:** 2025-02-03

**Authors:** Vladan Djordjević, Dmitar Lakušić, Ivan Novković, Vladimir Stevanović, Spyros Tsiftsis

**Affiliations:** 1 Institute of Botany and Botanical Garden, Faculty of Biology, University of Belgrade, Takovska 43, 11000 Belgrade, Serbia; dlakusic@bio.bg.ac.rs; 2 Faculty of Geography, University of Belgrade, Studentski trg 3, 11000 Belgrade, Serbia; ivan.novkovic@gef.bg.ac.rs; 3 Serbian Academy of Sciences and Arts, Kneza Mihaila 35, 11000 Belgrade, Serbia; 4 Department of Forest and Natural Environment Sciences, Democritus University of Thrace, 1st km Dramas-Mikrochoriou, 66100 Drama, Greece; stsiftsis@neclir.duth.gr

**Keywords:** altitude, Balkan Peninsula, belowground organs, climatic factors, distribution, geological substrates, habitat types, Orchidaceae, species richness

## Abstract

The Balkan Peninsula is considered one of the most important centres of orchid diversity in Europe. However, the patterns of orchid species richness in the Central Balkans have not been sufficiently studied so far. The aim of this study was, therefore, to identify the centres of orchid diversity and the factors that influence the spatial variation in orchid species richness in the Central Balkans. For the analyses, the area of the Central Balkans was divided into 10 × 10 km grid cells. The environmental variables determined for each grid cell and used in the analyses were altitude, bioclimatic variables, geological substrates and habitat types. A random forest (RF) analysis was used to identify the environmental predictors most strongly associated with species richness. In addition to the total number of taxa, orchids with three belowground organ types were analysed separately: (a) rhizomatous orchids, (b) orchids with palmately lobed and fusiform tubers (“palmate tuberous orchids”) and (c) orchids with spherical or ovoid tubers (“ovoid tuberous orchids”). In the Central Balkans, 54 orchid species and subspecies have been recorded, and the most important centres of diversity are the Tara, Zvijezda, Jadovnik and Zlatar Mountains and the Ovčar-Kablar Gorge. In general, two groups of grid cells with the largest number of orchid taxa, i.e., hotspots, stood out: (1) grid cells with a large altitudinal range and (2) grid cells occupied by gorges and ravines. The most important gradients influencing orchid species richness are specific habitat types and altitudinal ranges, while climatic factors and geological substrates are less important. The most important factors affecting the richness of total and rhizomatous orchids are altitudinal range and habitat types (*Abieti-Fagenion*, *Ostryo-Carpinion orientalis* and *Pinion nigrae* forests), highlighting the important role of habitat heterogeneity. The maximum altitude, percentage of *Abieti-Fagenion* and *Vaccinio-Picetea* forests and the minimum value of the mean temperature of the driest quarter are the most important factors for determining the richness of palmate tuberous orchids, whereas the percentage of xero-thermophilous habitat types (*Ostryo-Carpinion orientalis*, *Asplenietea trichomanis* and *Pinion nigrae*) has the greatest influence on the richness of ovoid tuberous orchids. These results confirm the hypothesis concerning the origin and development of underground organs in orchids, emphasising that palmate tuberous orchids are best adapted to cold and humid habitat conditions, whereas ovoid tuberous orchids have the ability to grow in habitats with very warm and dry conditions. This study provides a good basis for better orchid conservation planning and underlines the importance of belowground strategies as a feature of orchid life history that should be considered when studying patterns of orchid diversity.

## 1. Introduction

With 880 genera and 27,800 species and subspecies, the family Orchidaceae is one of the largest plant families in the world [1,2]. The family has a cosmopolitan distribution and includes the highest number of endangered species and genera, mainly due to habitat loss, changing environmental conditions such as climate change, land-use practices or their natural rarity [3]. The sensitivity of orchids is primarily attributed to their specific biology, which includes mycorrhizal associations, specific pollination systems and limited germination rates [4,5,6,7]. Due to their threat status and important role in ecosystems, orchids are often used as a flagship group for biological conservation [8]. Understanding the factors that determine the diversity of orchid species is fundamental for ecological and biogeographical research. In addition, studying their spatial distribution and diversity patterns helps to identify areas of high importance for orchid conservation [9] and provides better insight into ecologically designed plans for their conservation.

The diversity patterns of orchids in large geographical areas are primarily determined by the evolutionary and migratory history, macroclimatic factors and the latitude and size of the area [8,10,11,12,13,14,15,16], while at the regional level, the distribution of orchids is influenced by the physico-chemical properties of the soil, geological substrates, light regime, meso- and microclimates, altitude, habitat types and biotic factors, i.e., the specificity of mycorrhizal fungi and pollinators as well as the disturbance regime [17,18,19,20,21,22,23,24,25]. However, the importance of these factors varies from region to region, and there is no universal diversity pattern that applies to all orchids and regions. Studies have shown that on islands, for example, the size of the area and the maximum altitude are positively correlated with the number of orchid taxa [26]. Furthermore, the study of protected areas has found that their size significantly influences the richness of orchid species, especially in Africa, Asia and North America [14]. In contrast, the size of protected areas in Europe has less influence, as many species thrive in unprotected areas (e.g., regularly mown meadows or unprotected forests) [14,27]. Studies on the spatial distribution and abundance of orchids have mainly been conducted in Asia, Africa, Australia and America [11,12,13,28,29,30], whereas studies on orchid diversity have become increasingly common in Europe in recent years [23,31,32,33,34,35,36,37].

Some studies have shown that the diversity patterns of orchids are determined by characteristic features of their way of life, in particular by the presence of different pollination systems [32,36] and life forms [31,34,38,39,40]. It is known that the tropics and subtropics are centres of orchid diversity, as orchids with different life forms (epiphytes, terrestrial orchids and lithophytes) occur there. In Europe, orchids are exclusively terrestrial and inhabit almost all terrestrial ecosystems. The evolutionary development of the belowground organs of terrestrial orchids proceeded from rhizomes to spherical or ovoid tubers [41,42]. According to the concept explained in detail by Averyanov [41], Tatarenko [42] and Dressler [43] and categorised by Tsiftsis et al. [31] and Štípková et al. [34], terrestrial orchids can be divided into three main groups: (a) rhizomatous, (b) “intermediate orchids” or “palmate tuberous orchids”, i.e., orchids with palmately lobed and fusiform tubers (in an evolutionary sense, an intermediate stage between rhizomatous and ovoid tuberous orchids), and (c) “tuberous orchids” or “ovoid tuberous orchids”, i.e., orchids with spherical or ovoid tubers. The studies on the spatial variation in orchid richness, which include comparative analyses of the three orchid groups mentioned above, were carried out in Greece [31] and the Czech Republic [34]. However, the role of different belowground organ types of orchids in other regions of Europe remains to be investigated.

The taxonomic diversity of the family Orchidaceae gradually increases from northern to southern Europe and reaches its peak in the Mediterranean regions [44]. The most important centre of orchid diversity in Europe is the Eastern Mediterranean area, i.e., the area around the Aegean Sea [44,45]. Other areas in the Balkan Peninsula, the Alps, Sicily and the Caucasus are also important centres of diversity [44]. Although the Balkan Peninsula is recognised as an important centre of orchid diversity, the variation in orchid species richness at a spatial scale of 10 km grid cells and the influence of factors on patterns of orchid diversity have not been studied in the Central Balkans. In this region, patterns of orchid abundance have been studied, which refers to the variation in the population size of orchids, especially in grassland and herbaceous wetlands [21,46] and forest ecosystems [22]. Although the influence of environmental factors on orchid diversity patterns can be observed individually (e.g., climatic factors), in reality, there is a joint effect of several environmental factors [47].

The aims of this study were: (i) to determine the presence of orchid taxa in the area of the Central Balkans; (ii) to determine the orchid species richness in individual 10 × 10 km grid cells in the Central Balkans; (iii) to determine hotspots, i.e., grid cells with the largest number of orchid taxa; and (iv) to identify which factors (altitude, climatic factors, habitat types and geological substrates) have the greatest influence on orchid species richness. The patterns of species richness and distribution were analysed for the total orchid flora as well as for the orchids of specific belowground organ types (rhizomatous, palmate tuberous and ovoid tuberous orchids). The results of this study could help us to prioritise conservation and define specific conservation measures for the orchid groups studied.

We hypothesised that altitude, specific habitat types and individual climatic factors are the most important factors influencing patterns of orchid species richness in the Central Balkans. In addition, we assumed that the significant presence of carbonate bedrock types has an important influence on orchid richness, as shown in previous studies [23,31]. We hypothesised that the grid cells with the greatest altitudinal range (indicating habitat heterogeneity) and with the most diverse habitat types and predominance of carbonate bedrock types, would be the most important hotspots for orchids. We also hypothesised that patterns of orchid richness would differ depending on belowground organ types. Based on the hypotheses put forward by Averyanov [41], we assumed that the richness of palmate tuberous orchids would be greatest in the coldest and wettest areas, while most species with spherical or ovoid tubers would be found in the warmest and driest areas.

## 2. Materials and Methods

### 2.1. Study Area

**Figure 1:**
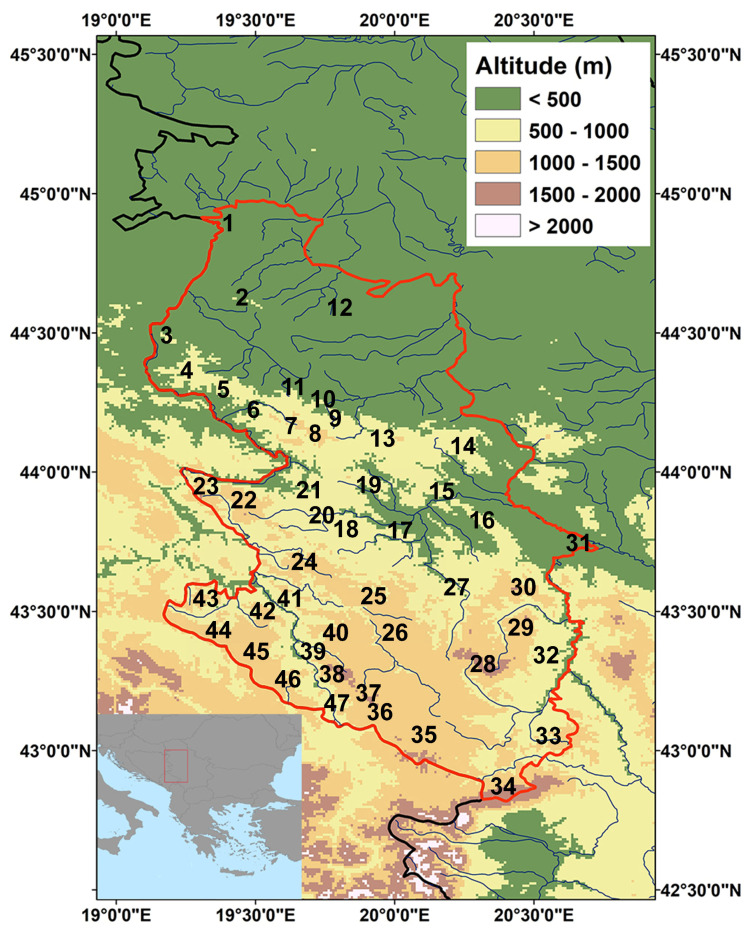
Map of the study area (Central Balkans: western part of Serbia) (the borders of the study area are marked with a red line) with the name of main localities: (1) Zasavica, (2) Mt Cer, (3) Mt Gučevo, (4) Mts Boranja and Jagodnja, (5) Mt Sokolska planina, (6) Azbukovica, (7) Mts Jablanik and Medvednik, (8) Mt Povlen, (9) Gradac river gorge, (10) Valjevo, (11) Vlašić, (12) Vladimirci, (13) Mt Maljen, (14) Mt Suvobor, (15) Ovčar-Kablar gorge, (16) Mt Jelica, (17) Mt Blagaja, (18) Mt Drežnička Gradina, (19) Kalenići, Bjeloperica, (20) Užice, (21) Mt Jelova Gora, (22) Mt Tara, (23) Mt Zvijezda, (24) Mt Zlatibor, (25) Mt Mučanj, (26) Mts Javor and Čemernica, (27) Ivanjica, (28) Mt Golija, (29) Mt Radočelo, (30) Mt Čemerno, (31) Kraljevo, (32) Raška (Brvenica), (33) Mt Rogozna, (34) Mt Mokra Gora (Prokletije), (35) Pešter, (36) Mt Giljeva, (37) Mt Ozren, (38) Mt Jadovnik, (39) Mileševka river gorge, (40) Mt Zlatar, (41) Priboj (Crni vrh), (42) Mt Pobijenik, (43) Mt Gajeva planina, (44) Bučje, (45) Jabuka, (46) Mt Kamena Gora, and (47) Brodarevo, Lim river gorge.

The study area is located in the north-central part of the Balkan Peninsula (42°50′–44°58′ N, 19°09′–20°39′) and covers approximately 18,000 km^2^ (Figure 1). It encompasses the western part of the Republic of Serbia, including the southern part of the Pannonian Plain in the north and the mountainous region of the Dinaric Alps in the central and southern areas. The altitude ranges from 69 m to 2154 m. The climate in this area can be characterised as temperate-continental with more or less pronounced local characteristics. In the mountains of western and southwestern Serbia, on the other hand, a humid mountain climate of the alpine type prevails. The average annual air temperature varies between 7.2 °C in the coldest region (Sjenica) and 12.2 °C in the warmest region (Loznica). In the mountainous areas above 1000 m, the average annual temperature is around 6.0 °C, while it is 3.0 °C in the areas above 1500 m. The relative humidity varies between 74.4% (Valjevo and Loznica) and 79.1% (Požega), while the annual precipitation varies between 749 mm in the lowlands and 1500 mm in the mountains of southwestern Serbia (data from the Hydrometeorological Service of the Republic of Serbia).

The study area is characterised by a great diversity of geological substrates, which is reflected in significant occurrences of various sedimentary, igneous and metamorphic rocks. A special feature is the high prevalence of carbonate bedrock types, ultramafic rocks, various types of siliceous rocks and ophiolitic mélanges [48,49,50]. There are several zonal vegetation types in the study area: alluvial forests (*Fraxino-Quercion roboris*), oak forests (*Quercion confertae* and *Quercion petraeo-cerridis*) (at low to medium altitudes), and beech and hornbeam forests (*Fagion sylvaticae* and *Carpinion betuli*) (mainly in the mountainous areas), while coniferous forests (*Vaccinio-Piceetea*) occur at high altitudes [51,52]. Among the forest vegetation types, the occurrence of these forests should be emphasised: forests occurring mainly in limestone canyons and gorges (*Fraxino orni-Ostryion*); mixed beech-fir forests (*Fagion sylvaticae*); pine forests occurring mainly in limestone canyons and gorges (*Fraxino orni-Pinion nigrae*); pine forests on ultramafics (*Erico-Fraxinion orni*); Scots pine forests distributed mainly on carbonate geological substrates (*Seslerio rigidae-Pinion*); forests of *Picea omorika* (*Erico carneae-Piceion omirikae*); mixed forests of pines, spruces and firs, mainly on calcareous mountain plateaus (*Dicrano-Pinion sylvestris*) [51,52,53]. The herbaceous vegetation types include grasslands, meadows, tall-herb vegetation, swards (*Molinio-Arrhenatheretea*, *Festuco-Brometea*, *Nardetea strictae*, *Juncetea trifidi* and *Mulgedio-Aconitetea*), bog and fen vegetation (*Scheuchzerio palustris-Caricetea fuscae*) and marshland vegetation (*Phragmito-Magnocaricetea*) [51].

### 2.2. Data Collection

The orchid database contains data on 56 orchid species and subspecies, which were recorded at 3692 localities and in 145 10 × 10 km grid cells in the universal transverse mercator projection. The dataset includes 53 orchid species and subspecies from 2716 localities collected during fieldwork between 1995 and 2024. In addition, the dataset contains data on 44 taxa from 292 localities (collected between 1850 and 2023) collected in the Herbarium of the Natural History Museum in Belgrade (BEO) and the Herbarium of the University of Belgrade (BEOU), as well as the published data on 49 species and subspecies from 684 localities (published between 1884 and 2024). The study is primarily based on the recent data obtained through our own fieldwork. The overall dataset contains only a few very old published data and data from herbaria. However, all these old data were confirmed by our fieldwork. Exceptions are the two species *Herminium monorchis* and *Orchis spitzelli* (one record per species), which were not found during the fieldwork. As the data on their occurrence in the study area are very old, we did not include these two species in the numerical analyses. The majority of the literature data and herbarium material dates from the last 40 years. The analyses in this study are, therefore, based on the existence of 54 orchid taxa (species and subspecies).

The orchid taxa were identified according to Baumann et al. [54] and Delforge [55], whereas the nomenclature follows POWO [56] and Djordjević et al. [57]. Hand-held GPS devices, the Garmin eTrex 30 and 32 in WGS 84, were used to determine the geographic coordinates (longitude and latitude) and altitude during fieldwork. Data from the herbarium collections and published sources were georeferenced using Ozi Explorer 3.95.4s software. A spatial unit of 10 × 10 km was chosen based on similar studies on orchid diversity [31,34]. We used a combined sampling method using a strip transect survey method (10 m wide and 1000 m long) and random sampling of orchid occurrence within each grid cell. Individual grid cells were visited in the sense of visiting a large number of different habitats and altitudinal zones. The minimum distance between two sampled sites with the same ecological conditions (same habitat type and geological substrate) was approximately 250 m. However, the minimum distance between sampling sites was shorter if the sites differed in their characteristics (if they were characterised by different habitats and geological substrates). An effort was made to investigate the grid cells with almost the same intensity. Exceptions were the grid cells on the borders of the study area with neighbouring countries or other regions of Serbia, as well as grid cells that included urban areas or predominantly agricultural fields. Due to the relatively small size of the study area and the long duration of the study, we are sure that the number of sites we missed during the search is negligible and, therefore, cannot have affected the outcome.

In addition to analysing the entire orchid flora, the orchids of the specific belowground organ types were also examined separately. According to the classification explained by Tsiftsis et al. [31] and Štípková et al. [34], terrestrial orchids are categorised into three groups based on the characteristics of their belowground organs: (a) rhizomatous, (b) palmate tuberous and (c) ovoid tuberous orchids.

For the analyses, the area of the Central Balkans (western part of Serbia) was divided into 10 km grid cells based on the military grid reference system (MGRS) and the universal transverse mercator (UTM) projection [58]. The environmental variables (predictors), determined for each grid cell and subsequently used in the analyses, were altitude, bioclimatic variables, geological substrates and habitat types. The bioclimatic variables for each 10 × 10 km grid cell were calculated using the WorldClim data with a 30 s resolution (approximately 1 km^2^) [59]. As the grid cells used in the present study (10 × 10 km grid cells) are not congruent with the 5 or 10 min raster data in this database, all bioclimatic variables (19 variables were downloaded in total) were aggregated by calculating the minimum, maximum and average values of the 30 s cells falling within each 10 × 10 km grid cell (57 variables in total). For altitude, the minimum, maximum, average and altitudinal range (maximum, minus and minimum value) were calculated (4 variables in total).

The geological maps of Serbia (https://geoliss.mre.gov.rs/prez/OGK/RasterSrbija/, accessed 16 November 2024) and the Generalized Habitat Map of Serbia [60,61] were used to extract the geological substrates and habitat types for the study area, respectively. Based on these maps, the percentage of each geological substrate and habitat type was calculated for each 10 × 10 km grid cell according to their representation. In total, 9 geological substrates and 19 habitat types were used. Specifically, the following bedrock types were used: (a) acidic and intermediate igneous rocks, (b) carbonate and silicate clastics, (c) flysch, (d) limestone and dolomite, (e) metamorphic rocks, (f) ophiolitic mélanges and Paleozoic clastics, (g) Quaternary sediments, (h) serpentine/ultramafics and (i) Tertiary silicate clastics and volcaniclastic/pyroclastic rocks. The following habitat types, listed through the associative names of vegetation units, were used for the analyses: (a) *Salicion*, (b) *Quercion roboris*, (c) *Quercion frainetto*, (d) *Ostryo-Carpinion orientalis*, (e) *Fagion sylvaticae*, (f) *Pinion nigrae*, (g) *Vaccinio-Piceetea*, (h) *Abieti-Fagenion*, (i) *Pino-Quercion*, (j) *Salicetea purpureae*, *Crataego-Prunetea* and *Sambuco-Salicion*, (k) *Vaccinion myrtilli-uliginosi*, (l) *Pinion mugo*, (m) *Juniperion nanae*, (n) *Festuco-Brometea*, (o) *Molinio-Arrhenatheretea*, (p) *Festuco-Seslerietea*, (q) *Asplenietea trichomanis*, (r) *Scheuchzerio-Caricetea fuscae* and (s) *Phragmito-Magnocaricetea* (Table S1). The names of the habitat types in this study are informal and associative and only partially correspond to the formal names of accepted syntaxa. All habitat types were grouped under these associative names of vegetation types, which are shown as separate areas in the Generalized Habitat Map of Serbia (Table S1) [60,61].

### 2.3. Data Analysis

To account for multicollinearity between the bioclimatic variables and altitude (61 variables in total), Pearson correlation coefficients were calculated for all pairwise interactions. To eliminate correlated variables, only one variable was selected among any of the pairs with a correlation coefficient r > |0.65|. Finally, out of the 61 climatic variables, only 7, consisting of maximum elevation, altitudinal range, maximum values of isothermality (max BIO3), maximum values of the mean temperature of the wettest quarter (max BIO8), maximum and minimum values of the mean temperature of the driest quarter (max and min BIO9) and minimum values of precipitation of the warmest quarter (min BIO18), remained for further analyses along with nine geological substrates and 19 habitat types.

A random forest (RF) analysis was conducted to identify the environmental predictors most strongly associated with species richness. A random forest (RF) model was chosen due to its advantages, including minimal parameter tuning requirements, no reliance on the specific distributions of explanatory variables, and the ability to handle non-linear relationships between variables [62]. The RF model provides variable importance rankings based on the mean square error (IncMSE) and the increase in node purity (IncNodePurity) metrics. Both of these metrics are used to determine which predictors most substantially explain the variations in species richness. IncMSE refers to the rise in the model’s error, estimated as the difference between the original error and the error observed when the variable’s values are randomised. Higher values of IncMSE indicate more important variables. IncNodePurity refers to the degree of the variable’s influence on each node of the decision tree. Higher IncNodePurity values indicate variables that contribute more substantially to reducing model impurity, thus identifying predictors with a stronger association with species richness. Here, we used the metric IncNodePurity to determine the most important variables [63].

To further explore and visualise the relationships between species richness and the most important environmental predictors identified in the random forest analysis, we utilised a generalised linear model (GLM) with a logit link function. This model served to illustrate the patterns and potential trends between species richness and each one of the most important predictors. As a measure of species richness, we used total species richness (including all orchid taxa of the study area) as well as sums of ovoid tuberous, palmate tuberous and rhizomatous orchids.

All analyses were performed in R version 4.4.1 [64], whereas the variable extraction was done using ArcGIS 10.8 [65].

## 3. Results

### 3.1. Distribution of Orchid Species Richness in the Central Balkans

The presence of 54 orchid species and subspecies in the Central Balkans (western part of Serbia) was established (Table 1). The analysis revealed that ovoid tuberous orchids dominate (20 species and subspecies), followed by rhizomatous orchids (19 species) and palmate tuberous orchids (15 species and subspecies).

**Figure 2:**
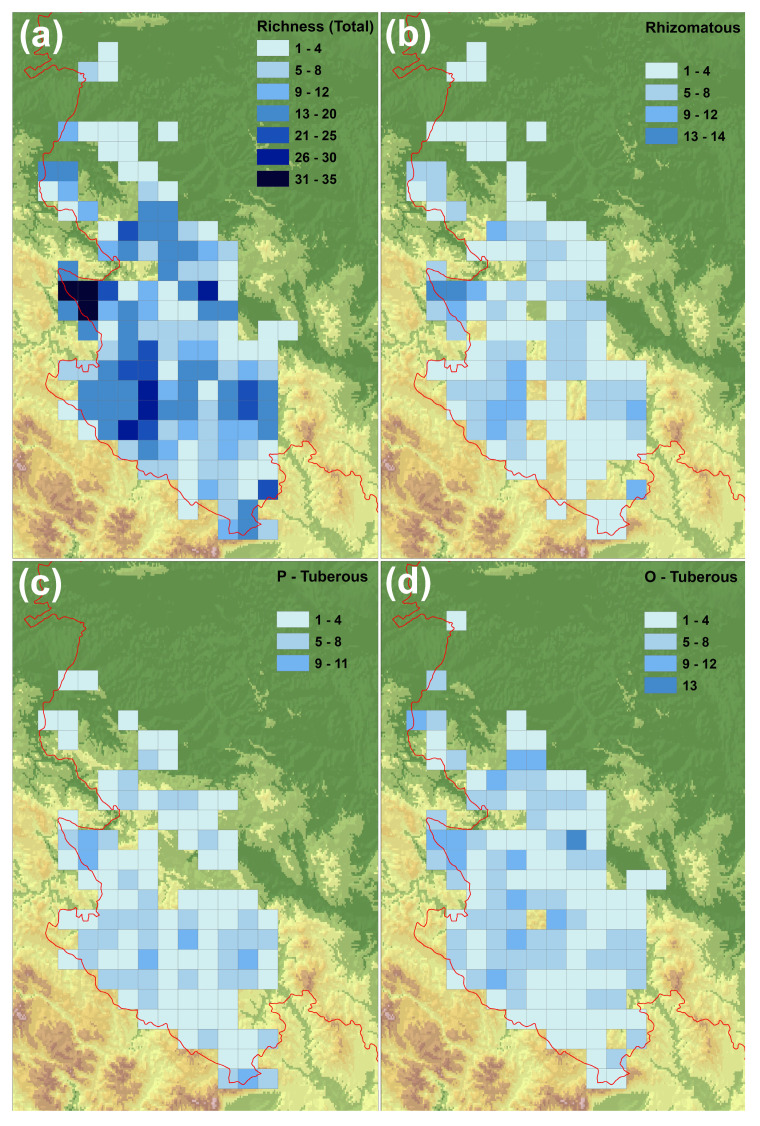
Distribution of orchid species richness in the Central Balkans (western part of Serbia): (**a**) total number of orchid taxa, (**b**) rhizomatous orchids, (**c**) palmate tuberous orchids and (**d**) ovoid tuberous orchids.

Orchids were recorded in 145 10 × 10 km grid cells in the Central Balkans (western part of Serbia) (Figure 2a). Ovoid tuberous orchids (Figure 2d) are the most widespread orchid group, present in 128 grid cells (88.28% of the total number of grid cells), followed by rhizomatous orchids (recorded in 120 grid cells; 82.76% of the total number of grid cells; Figure 2b) and palmate tuberous orchids (distributed in 110 cells; 75.86% of the total number of grid cells; Figure 2c).

The most orchid-rich areas of the Central Balkans are the Tara and Zvijezda Mountains in the western part, followed by the Jadovnik and Zlatar Mountains in the southwestern part and the Ovčar-Kablar Gorge in the eastern part of the study area (Figure 2a). In terms of the overall orchid floristic richness, 3 (2.07% of the total of 145) grid cells in the Central Balkans were identified as the richest (with 31–35 taxa), 4 (2.76%) grid cells were with 26–30 taxa, 9 (6.21%) grid cells were with 21–25 taxa, 40 (27.59%) grid cells were with 13–20 taxa, 19 (13.10%) grid cells were with 9–12 taxa, 30 (20.68%) grid cells were with 5–8 taxa and 40 (27.59%) grid cells were with 1–4 taxa (Figure 2a).

The rhizomatous orchids are mainly found in the Tara and Zvijezda Mountains in the western part, followed by the Jablanik and Medvednik Mountains in the northwestern part, the Zlatibor (Murtenica), Zlatar and Jadovnik Mountains and the Mileševka river gorge in the southwestern part, and the Rogozna Mountain and the Golija Mountain and Raška (Brvenica) in the southeastern part of the study area (Figure 2b). In terms of rhizomatous orchid richness, 2 (1.67% of the total 120) grid cells in the Central Balkans were identified as the richest (with 13–14 taxa), 10 (8.33%) grid cells were with 9–12 taxa, 40 (33.33%) grid cells were with 5–8 taxa and 68 (56.67%) grid cells were with 1–4 taxa (Figure 2b).

The palmate tuberous orchids are mainly found on Tara Mountain in the western part and the Javor and Čemernica Mountains in the south-central part, followed by the Golija and Radočelo Mountains in the eastern part, the Zlatar Mountain in the southwestern part, and Mokra Gora (Prokletije) in the southeastern part of the study area (Figure 2c). In terms of the richness of palmate tuberous orchids, 6 (5.45% of the total of 110) grid cells in the Central Balkans were with 9–11 taxa, 34 (30.91%) grid cells were with 5–8 taxa and 70 (63.64%) grid cells were with 1–4 taxa (Figure 2c).

Most ovoid tuberous orchids are found in Ovčar-Kablar Gorge in the eastern part, followed by the Tara and Zvijezda Mountains in the western part, Mts Jadovnik, Zlatibor (Gostilje) and Zlatar in the southwestern part, the Jablanik, Medvednik and Gučevo Mountains and the vicinity of Valjevo city in northwestern part, the Mučanj Mountain in the central part of the study area, and the Golija Mountain and Raška (Brvenica) in the southeastern part of the study area (Figure 2d). In terms of the richness of ovoid tuberous orchids, 1 (0.78% of the total of 128) grid cell in the Central Balkans was identified as the richest (with 13 taxa), 12 (9.38%) grid cells were with 9–12 taxa, 49 (38.28%) grid cells were with 5–8 taxa and 66 (51.56%) grid cells were with 1–4 taxa (Figure 2d).

### 3.2. Factors Determining Orchid Species Richness in the Central Balkans

The results of this study show that for each orchid group (total orchids, rhizomatous orchids, palmate tuberous orchids and ovoid tuberous orchids), four factors have the greatest influence on the distribution of orchid species richness in the Central Balkans according to the IncNodePurity metric (Table 2).

The most important factors affecting the total orchid species richness in each 10 × 10 km grid cell are, in order of importance, the percentage of *Abieti-Fagenion* forests, followed by the percentage of *Ostryo-Carpinion orientalis* forests, the altitudinal range and the presence of pine forests (*Pinion nigrae*) (Table 2).

The factors affecting the species richness of rhizomatous orchids are the same as those affecting total orchid species richness but with different weights, i.e., in a different order. The most important factors are the percentage of *Ostryo-Carpinion orientalis* forests, the altitudinal range, then the percentage of *Abieti-Fagenion* forests, and finally, the percentage of *Pinion nigrae* forests (Table 2).

The most important factors determining the species richness of palmate tuberous orchids are the maximum altitude, the percentage of *Abieti-Fagenion* and *Vaccinio-Piceetea* forests and the minimum values of the mean temperature of the driest quarter (BIO9) (Table 2).

The factors that have the greatest impact on the species richness of ovoid tuberous orchids are the percentages of *Ostryo-Carpinion orientalis* forests, *Asplenietea trichomanis* vegetation, *Pinion nigrae* forests and *Molinio-Arrhenatheretea* grasslands (Table 2).

**Figure 3:**
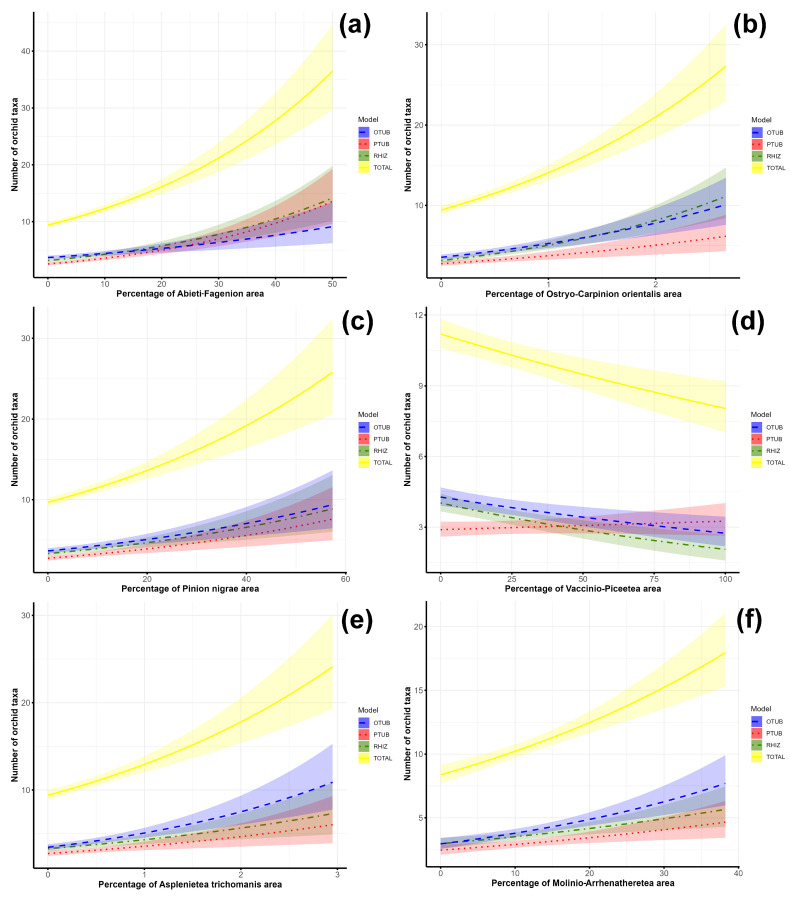
Association of species richness of orchids recorded in 10 × 10 km grid cells of the Central Balkans and selected habitat types: (**a**)—*Abieti-Fagenion*, (**b**)—*Ostryo-Carpinion orientalis*; (**c**) —*Pinion nigrae*; (**d**)—*Vaccinio-Piceetea*; (**e**)—*Asplenietea trichomanis*; (**f**)—*Molinio-Arrhenatheretea*. Shaded areas indicate ± 95% confidence intervals. (OTUB—ovoid tuberous orchids; PTUB—palmate tuberous orchids; RHIZ—rhizomatous orchids; TOTAL—total orchids).

The number of orchids (the total number of orchids and different orchid groups) increases when the percentage of the area covered by *Abieti-Fagenion*, *Ostryo-Carpinion orientalis*, *Pinion nigrae*, *Asplenietea trichomanis* and *Molinio-Arrhenatheretea* habitat types is increased (Figure 3a–c,e,f). However, orchid richness only increases when the percentage of *Abieti-Fagenion*, *Pinion nigrae* and *Molinio-Arrhenatheretea* accounts for about half of the area of each grid cell. The increased percentage of *Ostryo-Carpinion orientalis* and *Asplenietea trichomanis* habitat types leads to an increase in the number of species, but only up to a percentage of approximately 3%. In contrast to these habitat types, an increase in the area of *Vaccinio-Piceetea* forests in each grid cell leads to only a slight increase in palmate tuberous orchids, while other orchid groups decrease as the area of this habitat type increases (Figure 3d). However, it is noteworthy that orchid richness decreases when the grid cells are completely covered by this habitat type. In contrast, the richness of palmate tuberous orchids increases in the grid cells where the percentage of this habitat type is up to 100%.

**Figure 4:**
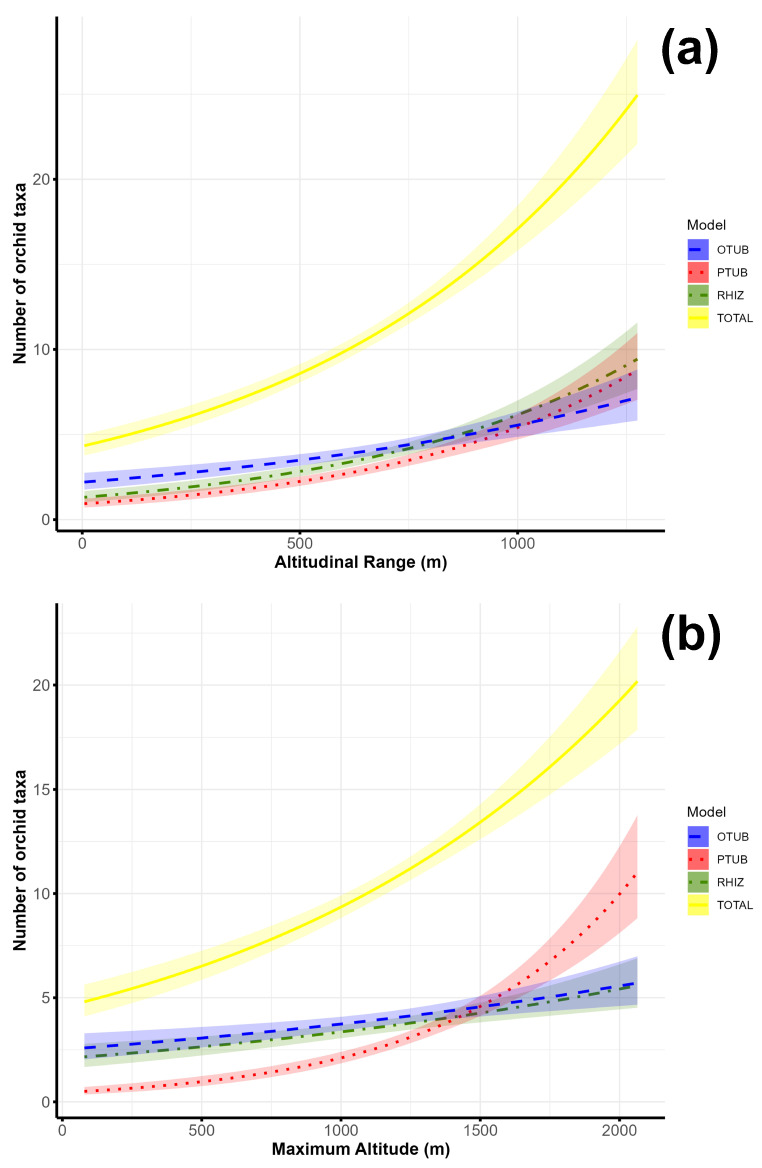
Association of species richness of orchids recorded in 10 × 10 km grid cells of the Central Balkans and selected altitudinal variables: (**a**)—altitudinal range; (**b**)—maximum altitude. Shaded areas indicate ± 95% confidence intervals. (OTUB—ovoid tuberous orchids; PTUB—palmate tuberous orchids; RHIZ—rhizomatous orchids; TOTAL—total orchids).

The number of orchids (the total number of orchids and different orchid groups) increases with an increasing altitudinal range (Figure 4a). Up to an altitude of 1000 m a.s.l., the ovoid tuberous and rhizomatous orchids dominate over palmate tuberous orchids, and above 1000 m a.s.l., the total number of species increases sharply, while the rhizomatous and palmate tubrous orchids dominate over ovoid tuberous orchids with increasing altitude range.

The number of orchids (the total number of orchids and different orchid groups) increases with an increasing maximum altitude (Figure 4b). The number of palmate tuberous orchids increases strongly above the maximum altitude of 1500 m a.s.l., while the number of ovoid tuberous and rhizomatous orchids gradually increases. Up to the maximum altitude of 1500 m a.s.l., ovoid tuberous and rhizomatous orchids dominate over the palmate tuberous orchids, and after the maximum altitude of 1500 m a.s.l., palmate tuberous orchids dominate over ovoid tuberous and rhizomatous orchids in terms of species number.

**Figure 5:**
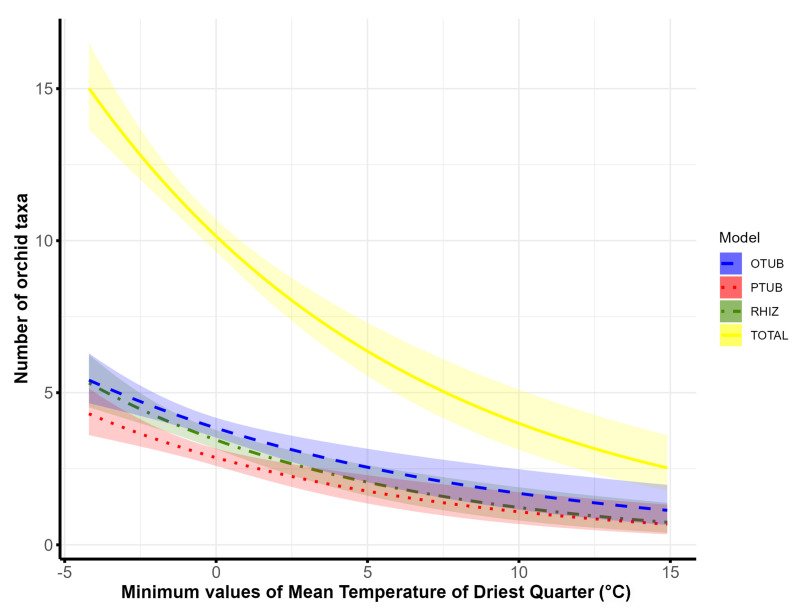
Association of species richness of orchids recorded in 10 × 10 km grid cells of the Central Balkans and minimum values of the mean temperature of the driest quarter (BIO9). Shaded areas indicate ± 95% confidence intervals. (OTUB—ovoid tuberous orchids; PTUB—palmate tuberous orchids; RHIZ—rhizomatous orchids; TOTAL—total orchids).

In contrast to the effects of altitudinal range on orchid richness, the number of orchids (the total number of orchids and different orchid groups) decreases with increasing minimum values of the mean temperature of the driest quarter (BIO9) (Figure 5).

## 4. Discussion

### 4.1. Distribution of Orchid Species Richness in the Central Balkans

The Balkan Peninsula is considered an important centre of vascular plant diversity [52,66,67]. However, the studies in the Central Balkans and the eastern Dinaric Alps have been neglected with regard to the spatial variations in orchid species richness. Therefore, this study contributes to the understanding of the diversity patterns of orchids in the Central Balkans and provides a better insight into the specificities of orchids with different underground organ systems.

In the Central Balkans, which includes the western part of Serbia, a total of 54 orchid taxa were recorded (73.97% of the total of 73 orchid taxa in Serbia) [57,68]. Among the Balkan countries, the largest number of orchids was recorded in Greece (193 taxa) [31,69,70] and Croatia (183 taxa) [71]. In Albania, 83 taxa—68 species and 15 subspecies—were recorded [72]; in Montenegro, approximately 80 taxa of orchids were recorded [73,74]; in Bosnia and Herzegovina, 76 taxa [75]; in Bulgaria, approximately 75 taxa [76]; in Slovenia, 73 taxa [77], and approximately 70 taxa in North Macedonia [78]. The lower number of orchid taxa in the Central Balkans (the western part of Serbia) can primarily be explained by the climatic characteristics of the region, i.e., the prevailing humid temperate-continental climate and the humid mountain climate of the alpine type. The lack of a Mediterranean climate and typical ecosystems of the Mediterranean areas, where most orchids of the genera *Ophrys*, *Orchis* and *Serapias* grow, also contributes to this pattern [55,69,70].

In general, the areas of high species richness in Serbia coincide with the areas of endemism of vascular plants [66]. Our research revealed that the greatest richness of orchid taxa is found in the mountainous regions (Mts Tara and Zvijezda in the western part and Mts Jadovnik and Zlatar in the southwestern part of the study area) (Figure 2a). This is in line with previous studies in Serbia, which showed that in the western part of Serbia, the greatest number of the Balkan endemic plant taxa occur on Mts Tara and Zvijezda [66]. However, our study emphasises the importance of other mountain areas that are not considered hotspots for endemic plant taxa, especially the Jadovnik and Zlatar Mountains. All areas (10 × 10 km grid cells) with the highest orchid species richness are characterised by the presence of gorges in the lowlands, mountain plateaus in the medium areas and the high mountain areas. In these grid cells, the altitudinal range is large, which indicates a great heterogeneity of habitats. The Tara and Zvijezda Mountains, for example, are rich in orchid species in the areas where the Drina, Derventa and Beli Rzav gorges are located in addition to the high mountain regions. The Jadovnik Mountain is rich in orchids in the parts where the Sopotnica valley, the Lim and Mileševka gorges and the high mountain areas are located. In addition, our study shows that large numbers of orchids have been observed in certain gorges and canyons as distinct entities (e.g., in the Ovčar-Kablar gorge, Gradac gorge, etc.), which highlights the importance of these refugial areas for the growth and survival of many members of the family Orchidaceae. In general, gorges and canyons in western Serbia are inhabited by the largest number of orchids of the sub-Mediterranean and Mediterranean chorological groups, which is consistent with the studies on the entire vascular flora of gorges and canyons in Serbia [53,73,79]. This distribution of centres of orchid diversity also corresponds to the general pattern of centres of plant diversity in montane coniferous forests in the central part of the Balkan Peninsula [52]. Namely, the areas with the greatest plant species richness in montane coniferous forests were recorded in mountainous areas with pronounced slopes, peaks, karst fields and mountain plateaus, as well as in deep gorges and canyons, which indicates that the most species-rich montane coniferous forests of the Central Balkans often grow in shallow soil and rugged terrain [52], which is also consistent with the pattern that rugged terrain tends to harbour more species-rich forests than flat or slightly hilly landscapes [80].

Our study revealed that the areas with low orchid richness are located in the northern part of the study area, which includes the lowlands, without large altitudinal ranges and with generally lower habitat heterogeneity. In addition, the low orchid richness in this area could also be due to intensive human impact (e.g., urbanisation, agriculture).

The present study shows that the patterns of orchid diversity differ depending on their belowground organ types. In the Central Balkans, ovoid tuberous orchids are the most widespread, followed by rhizomatous orchids and palmate tuberous orchids, which is in agreement with the results from Greece [31] and contradicts the results from the Czech Republic [34], which found that rhizomatous orchids are the most widespread species group, followed by palmate tuberous and ovoid tuberous orchids. The distribution patterns of orchids in the Central Balkans follow the transition from the Mediterranean climate of Greece to the continental climate of the Czech Republic. From Central to Southern Europe, the proportion of ovoid tuberous orchids, in particular, increases. Moreover, ovoid tuberous orchids are significantly less represented in the Central Balkans, comprising 37.04% of the total number of orchid taxa, compared to Greece, where they dominate with 71.50% of the total number of taxa [31].

The distribution of the richness of rhizomatous orchids in the Central Balkans (Figure 2b) is very similar to the distribution of total orchid richness (Figure 2a), and it is mainly in mountainous regions with rich forest vegetation, which corresponds to the fact that rhizomatous orchids grow mainly in forest ecosystems [55]. The greatest richness of palmate tuberous orchids (Figure 2c) is found in the high mountain regions, especially in the 10 × 10 km grid cells located on the highest peaks, which confirms the hypothesis that they are best adapted to cold and humid conditions [41]. In contrast, the greatest richness of ovoid tuberous orchids (Figure 2d) is found both in hilly mountainous regions, in gorges and canyons (e.g., Ovčar-Kablar gorge) and in the lowlands (around the towns of Užice and Valjevo), where thermophilous and mesophilous vegetation types predominate, confirming the claim that these orchids are best adapted to dry and warm environmental conditions [31,41].

### 4.2. Factors Determining Orchid Species Richness in the Central Balkans

It has been shown that climatic factors, which depend on the geographical location (latitude and longitude) and altitude of the area studied, play an important role in determining orchid richness at both macro and fine scales [8,12,26,31]. The role of geological substrates, soil properties and habitat types was neglected until recently, but they have now been shown to have a significant impact on orchid distribution, especially at regional and local scales [18,23,25,31,34,81]. Our study indicates that mainly habitat types and altitudinal factors influence the distribution of orchid taxa richness in the Central Balkans. Geological substrates and specific climatic factors, on the other hand, are not among the most important factors affecting the distribution of orchid richness.

#### 4.2.1. Factors Determining the Total Orchid Species Richness in the Central Balkans

The factors that most strongly influence the distribution of total orchid richness in the Central Balkans are the altitudinal range and the percentage of certain forest habitat types (Table 2). We found that the number of orchid taxa increases with the increasing altitudinal range of the grid cells, which is logical considering that areas with high altitudinal ranges (from the lowlands to high mountains) have a greater diversity of habitats and microhabitats and, thus, vegetation types. In particular, there is great variability in climatic conditions in areas with large altitudinal ranges, which, in turn, leads to greater heterogeneity in terms of the vegetation types that allow the survival of orchids with different environmental requirements.

In the Central Balkans, the overall richness of orchid taxa is particularly influenced by the percentage of the *Abieti-Fagenion* forest type, which is explained by the fact that the areas covered by this forest type are rich in both Central European and boreal orchid taxa. This forest type dominates in areas of medium and slightly higher altitudes and occurs on different geological substrates (both carbonate and siliceous and serpentine substrates) and enables the growth and survival of numerous orchids that are adapted to the mesophilous conditions of the habitat. Previous orchid research also indicates that many orchids and several species of genera *Epipactis* and *Cephalanthera* occur in beech and mixed beech forests [54,55,69,82,83,84]. It is important to emphasise that beech and mixed beech forests on the Balkan Peninsula have been identified as refugia for species of the genus *Epipactis*, making the entire Balkan region one of the most important centres of diversity of this genus in Europe [55,69].

On the other hand, the percentage of area occupied by *Ostryo-Carpinion orientalis* forests is another important factor affecting orchid species richness, which is not surprising considering that these thermo-mesophilous forests, mainly found in limestone gorges and canyons, are refugial forests and provide refuges for many vascular plant species [66], including representatives of the Mediterranean and sub-Mediterranean orchids, especially taxa of the genera *Orchis*, *Ophrys*, *Anacamptis* and *Himantoglossum*. Due to the frequent openness or semi-openness of forest stands, this habitat type is also inhabited by some orchids that grow mainly in grassland habitats. In western Serbia, 24 orchid taxa have been recorded in *Ostrya carpinifolia* forests, i.e., in communities of the alliance *Fraxino orni-Ostryon* [85], while in northeastern Greece, 22 orchid species have been recorded in communities of *Ostrya carpinifolia*, *Carpinus orientalis* and *Fraxinus ornus* [86].

*Pinion nigrae* forests also play an important role in determining the distribution of orchid species richness in the Central Balkans, which is explained by the significant presence of this type of vegetation, especially on serpentines, but also on the carbonate geological substrates of the study area. Due to high light intensity, this type of forest vegetation is often inhabited by orchids that prefer open grassland habitats and is therefore considered an alternative habitat for some typical grassland orchids [22].

Although the role of geological substrates in determining orchid species richness is not particularly emphasised at first glance, it can be assumed that the importance of bedrock types is largely overshadowed, i.e., covered by the importance of habitat types. Since the *Ostryo-Carpinion orientalis* forests occur predominantly on carbonate geological substrates [53], this habitat type indicates the importance of limestone, dolomite and carbonate clastites for orchid richness. This is consistent with previous studies that underline the importance of limestone and other carbonate geological substrates for the occurrence of terrestrial orchids [18,23,31,55,69,87,88]. However, in the study area, *Abieti-Fagenion* forests occur on carbonate bedrock types, ultramafics and siliceous substrates, while *Pinion nigrae* forests occur mainly on ultramafics, indicating the importance of ultramafics and siliceous substrates in influencing patterns of orchid diversity. It is also a fact that numerous orchid species in the Central Balkans grow on different geological substrates [21,22], indicating their plasticity and adaptability. Recent studies have shown that orchids tolerate substrates with different metal concentrations through their exclusion strategy [89,90].

#### 4.2.2. Factors Determining the Richness of Rhizomatous Orchids in the Central Balkans

The richness patterns of rhizomatous orchids are similar to those found for the total number of orchids in the Central Balkans, suggesting that areas with a large altitudinal range and a high percentage of forest habitat types (*Ostryo-Carpinion orientalis*, *Abieti-Fagenion* and *Pinion nigrae*) are particularly rich in rhizomatous orchids. This result is understandable considering that in the study area, with the exception of *Epipactis palustris*, all other rhizomatous orchids grow in forest vegetation types, so areas with large altitudinal ranges are very diverse in terms of the habitats that allow the survival and growth of rhizomatous orchids of different chorological groups. Among the rhizomatous orchids, those belonging to the boreal (*Corallorhiza trifida*, *Epipogium aphyllum*, *Goodyera repens* and *Neottia cordata*) and Central European chorological groups (*Epipactis leptochila* subsp. *neglecta*, *E. muelleri*, *E. pontica* and *E. purpurata*), which are rare or only recently found in the Central Balkans, should be emphasised [91,92,93].

In contrast to the Central Balkans, where altitudinal range is the most important factor, in Greece, maximum altitude was found to play the most important role in the distribution of rhizomatous orchids [31]. In contrast to Greece, where a Mediterranean climate prevails, and most Central European and boreal orchids grow at high altitudes, in the Central Balkans, due to the more continental climatic conditions, most rhizomatous orchids occur in areas of medium altitude [35].

The relationships of rhizomatous orchids to forest habitats are mainly reflected in the fact that many of them are holomycotrophic or partially mycoheterotrophic plants (e.g., *Limodorum abortivum*, *Neottia nidus-avis*, *Corallorhiza trifida* and *Epipogium aphyllum*) that are completely dependent on mycorrhizal symbionts, especially fungi that form ectomycorrhizal relationships with trees. Examples include *Neottia nidus-avis*, which specialises in fungi of the family Sebacinaceae that form ectomycorrhizas with trees [94]; *Epipactis microphylla*, which forms communities with ectomycorrhizal fungi of the genus *Tuber*, whose representatives form ectomycorrhizae with trees [95]; and *Limodorum abortivum*, which forms a relationship with ectomycorrhizal fungi of the genus *Russula* [96].

#### 4.2.3. Factors Determining the Richness of Palmate Tuberous Orchids in the Central Balkans

The most important factors affecting the richness of palmate tuberous orchids are the maximum altitude and the minimum values of the mean temperature of the driest quarter (BIO9), suggesting that low temperatures play an important role in determining the richness of this orchid group. Moreover, the percentages of *Abieti-Fagenion* and frigoriphilous *Vaccinio-Piceetea* forests indicate that the high mountain climate is favourable for the development of these orchids, especially considering that in the zone of the mentioned forests, fen habitats and wet meadows often occur, where most of the representatives of palmate tuberous orchids grow [46,55,97]. Our study is consistent with a study on orchids in Greece, which found that maximum altitude is the most important factor influencing the diversity patterns of palmate tuberous orchids [31].

Our results emphasise the importance of the minimum values of mean temperature in the driest quarter, suggesting that maintaining temperatures in the driest quarter, i.e., the summer months, may be crucial for the survival of these orchids. Air temperature is known to influence seedling germination and establishment, photosynthetic activity, flowering time, fruiting and the abundance and density of orchid populations [98,99,100,101]. In addition, species of the genus *Dactylorhiza* have been shown to require a vernalisation period to develop their leaves [102], in contrast to most ovoid tuberous species of the genera *Ophrys* and *Serapias*, which do not require a chilling period. Some studies have found that low temperatures, especially in the previous few seasons, are important for the development of palmate tuberous orchids. For example, the abundance of *Dactylorhiza maculata* and *Gymnadenia conopsea* at the northern limit of their distribution in Russia is negatively correlated with the temperature increase in the previous seasons, suggesting that lower temperatures are favourable for these orchids [103].

Our results are consistent with Averyanov’s hypothesis [41] that orchids with palmate and fusiform tubers tolerate cold and wet soils well, which is explained by the evolutionary development of these orchids. The author noted that the origin and development of the first orchids with palmate and fusiform tubers (when the first organ tubers are formed as nutrient reservoirs) is related to the Alpine orogenesis and the development of mountain habitats with low temperatures and that these orchids significantly expanded their distribution areas at the end of the Neogene and in the Pleistocene when temperatures were low.

In the Central Balkans, palmate tuberous orchids occurring exclusively or predominantly in high mountain regions stand out due to their high conservation value, such as the three subendemics of the Balkans and the Carpathians (*Gymnadenia frivaldii*, *Dactylorhiza cordigera* subsp. *cordigera* and *Dactylorhiza maculata* subsp. *transsilvanica*), as well as taxa that have a southern limit of their distribution in this part of Europe (*Dactylorhiza fuchsii* and *D. maculata* subsp. *maculata*) [46].

#### 4.2.4. Factors Determining the Richness of Ovoid Tuberous Orchids in the Central Balkans

The factors that have the greatest impact on the diversity patterns of ovoid tuberous orchids in the Central Balkans are the percentages of xerophilous and thermophilous habitat types such as *Ostryo-Carpinion orientalis*, *Asplenietea trichomanis* and *Pinion nigrae* as well as *Molinio-Arrhenatheratea* grasslands. Considering that vegetation is a strong indicator of habitat conditions, it is shown here that thermophilous and xero-mesophilous habitats are the most favourable for the development and survival of ovoid tuberous orchids. It can be concluded that areas (10 × 10 km grid cells) covering medium altitude areas with limestone gorges are indeed centres of diversity of ovoid tuberous orchids, especially of the Mediterranean and sub-Mediterranean chorological groups. Our results are consistent with Averyanov’s hypothesis [41] that ovoid tuberous orchids represent a terminal phase in the evolution of the underground organs of terrestrial orchids, which enable orchids to survive in warm and dry conditions.

*Asplenietea trichomanis*, which in the Generalized Habitat Map of Serbia includes both the vegetation of rocky crevices (*Asplenietea trichomanis*) and screes (*Thlaspietea rotundifolii*), represents the first stages of vegetation development. Both rocky habitats and rocky grasslands are frequently inhabited by ovoid tuberous orchids, as they can act as pioneer species, i.e., they colonise habitats where few other species occur and where there is little competition between plants. In addition, a large number of ovoid tuberous orchids grow in ecotones between rocky habitats and forest habitats. Ecotone zones are favourable for many ovoid tuberous orchids with weak competitive ability or high light requirements, as there is less competition for edaphic resources and the space is sufficiently illuminated [104,105]. Finally, many ovoid tuberous orchids are known to inhabit rocky grasslands (communities of the class *Festuco-Brometea*). This vegetation class is proven to be the richest in orchid species in the western part of Serbia [21,85]. Furthermore, “semi-natural dry grasslands and scrubland facies on calcareous substrates (Festuco-Brometalia)” are listed among the priority habitats listed in Annex I of the European Union Habitats Directive (92/43/EEC).

The forests of *Ostryo-Carpinion orientalis* and the *Asplenietea trichomanis* habitat types indirectly underline the important role of carbonate geological substrates for the diversity patterns of ovoid tuberous orchids, as they mainly occur on this geological substrate in the study area. This result is consistent with the results of studies in Greece, which have shown that the richness of ovoid tuberous orchid species increases with the increasing percentage of calcareous substrates in the grid cells in Greece and especially in the southern parts of the country [31].

The *Pinion nigrae* forests indicate the importance of serpentine areas for the growth and development of ovoid tuberous orchids, as these forests are most abundant on ultramafics. Ovoid tuberous orchids occur both in sparse *Pinion nigrae* forests and on serpentine steppes near or on the edge of these forests. Given the high occurrence of serpentine areas in the study area, which are not strongly influenced by agriculture and where competition between plants is known to be lower, they represent potential reserves and refuges for many ovoid tuberous orchids that are threatened by anthropogenic factors in areas with carbonate bedrock types [22].

Finally, the richness of ovoid tuberous orchids is largely determined by the percentage of meadow communities of the class *Molinio-Arrhenatheretea*, especially the meadow communities of the alliance *Arrhenatherion elatioris*. This vegetation type is particularly rich in orchids, not only in the Central Balkans but also in Central Europe [85].

The high conservation value of the orchid flora in the Central Balkans is reflected in the occurrence of the ovoid tuberous orchid *Himantoglossum calcaratum* subsp. *calcaratum*, which is endemic to the Balkans and grows mainly on carbonate geological substrates, on the edges of rocky habitats or along roadsides and forest edges, especially *Ostrya caprinifolia* and *Quercus* forests.

### 4.3. Implications for Conservation

Different richness patterns of orchid species of certain belowground organ types due to their different needs in relation to environmental factors have led to different strategies for their conservation. For the rhizomatous orchids, which mainly inhabit forest habitats, it is particularly important to maintain the diversity of forest habitats, especially at medium altitudes.

For the conservation of palmate tuberous orchids, it is particularly important to preserve the frigoriphilous vegetation of the high mountain sites of the study area and to maintain the minimum values of the mean temperature in the driest quarter. In the Central Balkans, the high mountain regions must be protected from degradation caused by the construction of tourist facilities and ski slopes. Tourism has a negative impact on the conditions of palmate tuberous orchids, especially in the centres of mountain tourism in western Serbia, in the immediate vicinity of catering facilities, hiking trails, ski resorts, sports grounds, huts and hotels, but also on the mountain peaks themselves. The negative effects of this factor can be seen in the disruption of the hydrological regime, the ruderalisation of ecosystems, the fragmentation and destruction of natural ecosystems in which orchids grow and pollution [106]. However, as palmate tuberous orchids occur only sporadically and rarely in the lowland areas of the Central Balkans, there is a high risk that their populations will drastically decline and disappear due to the effects of global warming [107]. The strong zoo-anthropogenic influences in the form of agriculture and urbanisation also contribute to the negative impact on this orchid group. Therefore, the conservation of low-altitude sites with habitats and microhabitats rich in palmate tuberous orchids is of great importance for their protection.

The conservation of ovoid tuberous orchids in the Central Balkans should focus primarily on the protection of medium- and low-altitude areas and limestone gorges. These orchids are endangered by the construction of hydropower plants and by zoo-anthropogenic impacts due to urbanisation, agriculture and pollution. In the Balkans, the construction of around 3000 hydropower plants is planned [108], which will seriously jeopardise the survival of ovoid tuberous orchids both directly and indirectly by changing the microclimatic conditions in the area.

Conservation measures include detailed studies on the in situ protection of orchids and their habitats using species distribution modelling as well as the identification of priority areas for the protection of orchids with different belowground organ types, i.e., work to identify potential reserves [3,9,109]. Within existing protected areas, a long-term monitoring programme for orchids and their population dynamics needs to be established. For all orchids, the promotion of sustainable agriculture without excessive use of pesticides and artificial fertilisers, as well as the development of ecologically sustainable tourism, maintenance of the hydrological regime of habitats and the application of citizen science involving the local population are necessary. In addition, the ex situ conservation of orchids, the cultivation of orchids in botanical gardens, the establishment of seed banks and the development of techniques for the germination and reintroduction of orchids are needed [110].

## 5. Conclusions

This study highlights the existence of two groups of areas with the largest number of orchid taxa, i.e., hotspots, in the Central Balkans: (1) grid cells with a large altitudinal range and (2) grid cells occupied by gorges. The results indicated that the specific habitat types and altitudinal range are the most important factors influencing the richness of orchid species, which emphasises the importance of habitat heterogeneity. The influence of climatic factors and geological substrates, on the other hand, proved to be less significant. This study showed that orchids with different belowground organ types have different richness patterns and gradients influencing them. It was found that palmate tuberous orchids favour high mountain regions with lower temperatures, ovoid tuberous orchids are most abundant at low and medium altitudes, especially in areas with gorges, while rhizomatous orchids are mainly found in forest areas. Future studies should clarify how the distribution patterns of orchids of individual underground organ systems change in response to climate change and land use. The role of the distribution of suitable mycorrhizal fungi and insect pollinators in determining the distribution patterns of orchids with particular belowground organ types should also be investigated.

## Figures and Tables

**Table 1:** Overview of orchids in the Central Balkans (western part of Serbia) with their specific belowground organ types: R—rhizomatous orchids; P-TUB—palmate tuberous orchids; O-TUB—ovoid tuberous orchids.

Orchid Taxon	Belowground Organ Types
*Anacamptis coriophora* (L.) R.M.Bateman, Pridgeon & M.W.Chase subsp. *coriophora*	O-TUB
*Anacamptis morio* (L.) R.M.Bateman, Pridgeon & M.W.Chase subsp. *morio*	O-TUB
*Anacamptis morio* subsp. *caucasica* (K.Koch) H.Kretzschmar, Eccarius & H.Dietr.	O-TUB
*Anacamptis palustris* subsp. *elegans* (Heuff.) R.M.Bateman, Pridgeon & M.W.Chase	O-TUB
*Anacamptis papilionacea* (L.) R.M.Bateman, Pridgeon & M.W.Chase subsp. *papilionacea*	O-TUB
*Anacamptis pyramidalis* (L.) Rich.	O-TUB
*Cephalanthera damasonium* (Mill.) Druce	R
*Cephalanthera longifolia* (L.) Fritsch	R
*Cephalanthera rubra* (L.) Rich.	R
*Coeloglossum viride* (L.) Hartm.	P-TUB
*Corallorhiza trifida* Châtel.	R
*Dactylorhiza cordigera* (Fr.) Soó subsp. *cordigera*	P-TUB
*Dactylorhiza fuchsii* (Druce) Soó subsp. *fuchsii*	P-TUB
*Dactylorhiza incarnata* (L.) Soó subsp. *incarnata*	P-TUB
*Dactylorhiza maculata* (L.) Soó subsp. *maculata*	P-TUB
*Dactylorhiza maculata* subsp. *transsilvanica* (Schur) Soó	P-TUB
*Dactylorhiza saccifera* (Brongn.) Soó subsp. *saccifera*	P-TUB
*Dactylorhiza sambucina* (L.) Soó	P-TUB
*Epipactis atrorubens* (Hoffm.) Besser	R
*Epipactis distans* Arv.-Touv.	R
*Epipactis helleborine* (L.) Crantz subsp. *helleborine*	R
*Epipactis leptochila* subsp. *neglecta* Kümpel	R
*Epipactis microphylla* (Ehrh.) Sw.	R
*Epipactis muelleri* Godfery subsp. *muelleri*	R
*Epipactis palustris* (L.) Crantz	R
*Epipactis pontica* Taubenheim	R
*Epipactis purpurata* Sm.	R
*Epipogium aphyllum* Sw.	R
*Goodyera repens* (L.) R.Br.	R
*Gymnadenia conopsea* (L.) R.Br.	P-TUB
*Gymnadenia frivaldii* Hampe ex Griseb.	P-TUB
*Gymnadenia odoratissima* (L.) Rich.	P-TUB
*Himantoglossum calcaratum* (Beck) Schltr. subsp. *calcaratum*	O-TUB
*Limodorum abortivum* (L.) Sw.	R
*Neotinea tridentata* (Scop.) R.M.Bateman, Pridgeon & M.W.Chase subsp. *tridentata*	O-TUB
*Neotinea ustulata* (L.) R.M.Bateman, Pridgeon & M.W.Chase	O-TUB
*Neottia cordata* (L.) Rich.	R
*Neottia nidus-avis* (L.) Rich.	R
*Neottia ovata* (L.) Bluff & Fingerh.	R
*Nigritella rhellicani* Teppner & E.Klein	P-TUB
*Ophrys apifera* Huds.	O-TUB
*Ophrys insectifera* L. subsp. *insectifera*	O-TUB
*Ophrys scolopax* subsp. *cornuta* (Steven) E.G.Camus	O-TUB
*Ophrys sphegodes* Mill. subsp. *sphegodes*	O-TUB
*Orchis mascula* subsp. *speciosa* (Mutel) Hegi	O-TUB
*Orchis militaris* L. subsp. *militaris*	O-TUB
*Orchis pallens* L.	O-TUB
*Orchis purpurea* Huds. subsp. *purpurea*	O-TUB
*Orchis simia* Lam. subsp. *simia*	O-TUB
*Platanthera bifolia* (L.) Rich.	P-TUB
*Platanthera chlorantha* (Custer) Rchb	P-TUB
*Pseudorchis albida* (L.) Á.Löve & D.Löve	P-TUB
*Spiranthes spiralis* (L.) Chevall.	O-TUB
*Traunsteinera globosa* (L.) Rchb.	O-TUB

**Table 2:** Variables’ importance based on their IncNodePurity scores. Not important variables are considered those classified in the bottom 50% of the IncNudePurity scores. The numbers next to the environmental factors indicate the order of their importance in the random forest model (first most important, second most important, third most important and fourth most important).

Environmental Factors		The Importance of Environmental Factors in the Random Forest Model			
		Total Number of Orchids	Rhizomatous Orchids	Palmate Tuberous Orchids	Ovoid Tuberous Orchids
Altitudinal factors	Altitudinal range	3	2	Not important	Not important
	Maximum altitude	Not important	Not important	1	Not important
Climatic factors	Minimum values of mean temperature of driest quarter (BIO9)	Not important	Not important	4	Not important
Habitat types expressed through associative names of vegetation units	*Abieti-Fagenion*	1	3	2	Not important
	*Ostryo-Carpinion orientalis*	2	1	Not important	1
	*Pinion nigrae*	4	4	Not important	3
	*Vaccinio-Piceetea*	Not important	Not important	3	Not important
	*Asplenietea trichomanis*	Not important	Not important	Not important	2
	*Molinio-Arrhenatheretea*	Not important	Not important	Not important	4

## Data Availability

The data presented in this study are available upon request from the corresponding author.

## Supplementary Materials

The following supporting information can be downloaded at: https://www.mdpi.com/article/10.3390/plants14030443/s1, Table S1: Detailed overview of habitat types in the study area according to Lakušić et al. [60,61].
