# Supplementary material for: Factors Influencing Orchid Species Richness in the Central Balkans: The Importance of Belowground Organ Types
Source: Plants (Basel). 2025 Feb 3;14(3):443. doi: 10.3390/plants14030443 (PMC11819906; doi:10.3390/plants14030443)
Supplement: Supplementary file 1 [file plants-14-00443-s001.zip › plants-3392275-supplementary.pdf]

Supplementary Table S1. Detailed overview of habitat types in the study area according to Lakušić et al. [60,61].

Associative phytocenological name	The final name of the cartographic unit	Corresponding habitat types according to the Rulebook on Habitats in Serbia	
Salicion	Hygrophilous willow and poplar forests	A1.1	*Salix alba* and *Populus* spp. forests
		A1.2	*Alnus* spp. and *Fraxinus angustifolia* forests
Quercion roboris	Hygrophilous forests of pedunculate oak and narrow-leaved ash	A1.3	*Quercus robur* and *Fraxinus angustifolia* forests
		A1.4	*Quercus robur* and *Carpinus betulus* forests
Quercion frainetto	Xerophilous oak forests	A2.1	*Quercus frainetto* and *Quercus cerris* forests
		A2.2	*Quercus pubescens* and *Quercus virgiliana* forests
		A2.3	*Quercus robur* and *Acer tataricum* forests
		A2.4	*Quercus trojana* forests
		A2.5	*Quercus petraea* and *Quercus cerris* forests
		A2.6	*Quercus petraea* and *Carpinus betulus* forests
		A2.7	*Quercus dalechampii* forests
		A2.8	*Quercus polycarpa* forests
Ostryo-Carpinion orientalis	Xerophilous oriental hornbeam and European hop-hornbeam forests	A2.9	*Carpinus orientalis* and *Ostrya carpinifolia* forests
		A2.A	*Acer monspessulanum* forests
Fagion sylvaticae	Mesophilious forests of beech and hornbeam	A3.1	*Carpinus betulus* forests
		A3.2	*Fagus sylvatica* forests
		A3.3	*Acer heldreichii* forests
Pinion nigrae	Thermophilous coniferous forests	A5.1	*Pinus nigra* and *Pinus sylvestris* forests
Vaccinio-Piceetea	Frigoriphilous coniferous forests	A6.1	*Picea* spp. and *Abies* spp. forests
		A6.2	*Pinus sylvestris* forests
		A6.3	*Pinus heldreichii* and *Pinus peuce* forests
Abieti-Fagenion	Mixed deciduous forests with spruces and firs	A8.1	Mixed deciduous forests with spruces (*Picea* spp.) and firs (*Abies* spp.)
Pino-Quercion	Mixed deciduous forests with pines	A8.2	Mixed deciduous forests with pines (*Pinus* spp.)
Salicetea purpureae	Broad-leaved hygrophilous shrubs	B1	Broad-leaved hygrophilous shrubs
Crataego-Prunetea	Broad-leaved xerophilous shrubs	B2	Broad-leaved xerophilous shrubs
Sambuco-Salicion	Broad-leaved mesophilous shrubs	B3	Broad-leaved mesophilous shrubs
Vaccinion myrtilli-uliginosi	High mountain heaths	B4.2	High mountain heaths
		B4.3	Alpine scrubs
Pinion mugo	Dwarf pine scrubs	B6.1	Dwarf pine scrubs (*Pinus mugo*)
		B6.3	Subalpine spruce scrubs (*Picea abies subalpina*)
Juniperion nanae	Juniperus scrubs	B6.2	Juniperus scrubs (*Juniperus nana*)
		B6.3	Subalpine spruce scrubs (*Picea abies subalpina*)
Festuco-Brometea	Central Balkan grassland	C1.3	Dry carbonate grasslands and rocky fields

	communities on rocky fields	C1.4	Dry serpentinite grasslands and rocky fields
		C1.5	Dry siliceous grasslands and rocky areas
Molinio-Arrhenatheretea	Mesophilous and wet grasslands	C2	Mesophilous grasslands
		C3	Seasonally wet and wet grasslands
Festuco-Seslerietea	Alpine and subalpine grassland formations	C4	Alpine and subalpine grassland formations
Asplenietea trichomanis	Rocks and screes	D2	Screes
		D3	Dry rocks and cliffs
		D4	Dry slopes and sections of loose material
		D5	Wet rocks and cliffs
		D7	Quarries
Scheuchzerio-Caricetea fuscae	Fens and bogs	E2	Poor fens
		E3	Rich fens
Phragmito-Magnocaricetea	Marshes	E4	Marshes
